# An examination of factors influencing the choice of therapy for patients with coronary artery disease

**DOI:** 10.1186/1471-2261-6-31

**Published:** 2006-07-04

**Authors:** Shashivadan P Hirani, Jonathan A Hyam, Shahzad Shaefi, John M Walker, Robin K Walesby, Stanton P Newman

**Affiliations:** 1 Health Psychology Unit, Centre for Behavioural and Social Sciences in Medicine University College London, Wolfson Building, 48 Riding House Street, London W1W 7EY, UK; 2 Centre for Cardiology and The Hatter Institute for Cardiovascular Studies University College London Hospital, Grafton Way, London WC1E 6DB, UK; 3 The Heart Hospital University College London Hospital, 16 Westmoreland Street, London W1G 8PH, UK

## Abstract

**Background:**

A diverse range of factors influence clinicians' decisions regarding the allocation of patients to different treatments for coronary artery disease in routine cardiology clinics. These include demographic measures, risk factors, co-morbidities, measures of objective cardiac disease, symptom reports and functional limitations. This study examined which of these factors differentiated patients receiving angioplasty from medication; bypass surgery from medication; and bypass surgery from angioplasty.

**Methods:**

Univariate and multivariate logistic regression analyses were conducted on patient data from 214 coronary artery disease patients who at the time of recruitment had been received a clinical assessment and were reviewed by their cardiologist in order to determine the form of treatment they were to undergo: 70 would receive/continue medication, 71 were to undergo angioplasty and 73 were to undergo bypass surgery.

**Results:**

Analyses differentiating patients receiving angioplasty from medication produced 9 significant univariate predictors, of which 5 were also multivariately significant (left anterior descending artery disease, previous coronary interventions, age, hypertension and frequency of angina). The analyses differentiating patients receiving surgery from angioplasty produced 12 significant univariate predictors, of which 4 were multivariately significant (limitations in mobility range, circumflex artery disease, previous coronary interventions and educational level). The analyses differentiating patients receiving surgery from medication produced 14 significant univariate predictors, of which 4 were multivariately significant (left anterior descending artery disease, previous cerebral events, limitations in mobility range and circumflex artery disease).

**Conclusion:**

Variables emphasised in clinical guidelines are clearly involved in coronary artery disease treatment decisions. However, variables beyond these may also be important factors when therapy decisions are undertaken thus their roles require further investigation.

## Background

Coronary artery disease (CAD) in the United Kingdom accounted for over 117,000 deaths during 2002 and continues to represent a common cause of mortality in the UK and in most developed societies [1]. Current management options for CAD fall into three main categories, namely medical therapy, percutaneous transluminal coronary angioplasty with or without stenting, (PTCA) and coronary artery bypass grafting (CABG). In the UK in the National Health Service up to 45,000 PTCA and 28,500 CABG are conducted annually; each at a current average cost of £2,609 (~$4,500) and £7,066 (~$12,000) respectively [2]. Where detailed guidelines on the use of PTCA and CABG have been proposed, they address a myriad of precise individual scenarios and are considered extremely complex [3-8]. Many parameters such as the variability of symptoms and employment status are not addressed in guidelines despite being considered to have an important role in decision making and being routinely addressed by clinicians. Rapid innovations in treatment often leave guidelines struggling to keep pace with current practice. In this context, the advent of minimally invasive options for surgery, advanced percutaneous techniques and improved medication regimens alter the risk benefit ratio and often result in guidelines lagging behind with technological advances that are already in clinical use.

A range of studies have reported that a not insignificant proportion of PTCAs and, to a lesser degree, CABGs are performed inappropriately or with uncertain appropriateness [9-11]. These figures vary according to location (e.g. US data indicates overuse of revascularization and UK data its under use), the reference guidelines utilised and the recency of the update of the guidelines. The allocation of patients to particular therapies appears to be influenced by a range of factors beyond guidelines and recommendations.

The aim of this paper was to examine the factors that influence clinical decisions regarding the allocation of patients to different treatments for coronary artery disease in routine cardiology clinics. It was hypothesised that clinicians would use both factors within guidelines as well as those outside the guidelines. Separate analyses were conducted to compare pairs of treatments from medication, PTCA and CABG, to enable stepwise analyses to be conducted that could determine the 'best' predictor variables. It was expected that for the more disparate interventions (e.g. medication and CABG), objective criteria would be likely predictors of treatment choice; but when interventions were less disparate (e.g. PTCA vs. CABG or medication vs. PTCA), variables beyond those in the guidelines would be likely to exert a predictive influence.

## Methods

### Patient population

Between January 1999 and February 2001, 294 patients with CAD from the caseloads of seven cardiologists, from three National Health Service centres in London and surrounding areas were selected as part of a convenience sample. The participants were invited to take part in the study in person or by mail. The patients were approached after their cardiologist/consultant determined the form of treatment they were to undergo, following a review of clinical data (including angiograms) and patient consultations. The clinician determined whether the patients were to start/continue medication or undergo revascularisation by PTCA or CABG surgery. Two-hundred and fourteen patients (72.79%) consented to take part in the study. The response rate per treatment was similar, with 73.00% of those about to undergo CABG, 74.74% of those about to undergo PTCA and 70.71% of those to receive medication consenting; with the resulting sample consisting of 70 in receipt of medication, 71 to receive PTCA and 73 to receive CABG surgery.

### Measures

Objective disease measures (angiogram data) were retrieved from patient medical records. Demographic, medical history, subjective symptoms and functional limitations data were obtained from patient self-reported responses to a questionnaire pack collated for the study. Demographic details included gender, age, employment situation, and educational level (scored on a scale from 1 – no formal education after compulsory level, to 4 – graduate/professional exams and above).

Previous cardiac history (previous interventions, previous myocardial infarction), coronary risk factors (hypercholesterolemia, hypertension, diabetes, smoking history and family history for myocardial infarction, ischaemic heart disease, stroke and neurological conditions) and co-morbidities (arthritis/rheumatism, respiratory disease, renal complications, cerebral events, gastrointestinal, peripheral vascular problems, thyroid problems and varicose veins) were measured as binary yes/no responses, except for smoking history which had three categories: never smoked, ex-smoker and still smoking.

Symptom reports were elicited for angina, dyspnoea and for a range of non-cardiac symptoms. Two items asked patients the frequency with which they currently experienced chest pains and shortness of breath. A further 31 items assessed a range of general symptoms (e.g. fatigue, sleep difficulties, muscle pains, vomiting) from which an average non-cardiac symptom-experience score was produced. Responses for all these items were recorded on a 4-point scale, from *all the time *to *never*, with higher scores representing greater frequency.

Scores for functional limitations due to cardiac symptoms were collected through scales adapted from Feinstein ratings [12]. These scales provided two scores relating to the level of functional impairment resultant from (i) angina and (ii) dyspnoea on the dimensions of task, pace and daily functions. Averaged impairment scores ranged from 0 to 4; higher scores represented greater limitations.

Further health related functional disability scores were obtained from the six subscales of the Sickness Impact Profile-68 (SIP) [13]. The scales were: 'social behaviour' (scores ranging from 0 to 12), 'mobility range' (0 to 10), 'emotional stability' (0 to 6),'somatic autonomy' (0 to 17), 'mobility control' (0 to 12) and 'psychological autonomy & communication' (0 to 11), with higher scores representing greater limitations in daily functioning.

An objective measure of disease severity was obtained from angiograms. These were analysed for the level of disease in three coronary disease prone arteries: the left anterior descending, the circumflex and right coronary arteries, each of which was rated on a four point scale (no significant disease, mild, moderate or severe). Additional measures of left ventricular function (no significant problems, mild, moderate or severe problems) and the number of diseased arteries were recorded, providing a total of five scores. These ratings were based on the agreed ratings of two cardiologists.

The majority of patients (86.9% – 186/214) patients were administered the questionnaires by one of the authors (SPH) at the bedside or in home interviews. However due to logistical reasons 5 medication and 23 PTCA patients completed the questionnaires by themselves and posted them back to the researchers.

Ethical approval for this study was granted by: Research and Development Directorate of University College London Hospital NHS Trust (ref: 99/0059) and the Clinical Research, Education, Effectiveness and Development Local Research and Ethics Committee of the Whittington Hospital (ref: 99/05L).

### Statistical analyses

The SPSS for Windows 11, LISREL 8.52 and SAS v8.2 statistical software packages were used for the analyses.

Preliminary examinations of the data revealed that the overall level of missing data was 3.43% (279 scores from 8132 items). Nevertheless, sixty cases (28.04%) had data missing on at least one variable, indicating that although the overall level of missing data is low, its effects can be relatively large. Missing values were imputed using the Markov Chain Monte Carlo algorithm through LISREL after finding no statistically reliable deviation from randomness using Little's MCAR test: χ^2 ^= 80.272, df = 96, p = 0.876 (within SPSS 11). The remaining analyses were conducted on the imputed data set [14,15].

Differences between treatment groups on each predictor variable was tested using the χ^2 ^test and one-way ANOVA's (Brown-Forsythe test when unequal variances) with post hoc Duncan's multiple range tests. Significance levels were set at p < 0.01. Where appropriate, measures of the strength of association between variables are provided through omega squared (ω^2^) and Cramer's phi (φ_c_), also see Table 1.

**Table 1:** Criteria for statistical interpretation

	Statistic		Interpretation
Strength of association	ω^2^	≈ 0.01	small association
		≈ 0.06	medium association
		≈ 0.14	large association
	
	φ_c_	< 0.3	little or no association
		0.3 to 0.7	weak association
		0.7 to 1.0	strong association

Regression model evaluation	deviance statistic	-	significance of unexplained (residual) variance in the dependent variable; thus is desired to be non-significant
	Nagelkerke's R^2^	-	the association between predictor variables and the outcome variable. Statistic ranges from 0 to 1 with higher scores indicating stronger associations.
	% of correctly classified cases	-	calculated at the start of the procedure and at the end of the analysis using the cut-off point of 0.5 for the estimated probability of the outcome variable
	sensitivity	-	the proportion of true positives or the proportion of cases correctly identified by the test as meeting the response category
	specificity	-	the proportion of true negatives or the proportion of cases correctly identified by the test as meeting the reference category
	c statistic	-	varies from 0.5 (the models predictions are no better than chance) to 1 (the model always assigns the higher probabilities to correct cases than incorrect ones)

Thereafter, three separate sets of logistic regression analyses were conducted with the same analytic procedures. In each analysis the likelihood of a particular treatment option (response category) was compared to a less invasive procedure (the reference category). The analyses performed were: (i) PTCA vs. medication, (ii) CABG vs. PTCA, and (iii) CABG vs. medication.

A series of univariate logistic analyses were conducted to identify individually significant predictors of the response treatment from the reference treatment in each of the three analyses. The likelihood ratio criterion was employed to test for the significance (p < 0.05) of each predictor. Variables with p < 0.05 within the univariate analyses were entered into a stepwise multivariate logistic analysis, to identify independent predictors of treatment type in each analysis. Significance was set at p < 0.01 in these multivariate logistic regressions. Variables within the regression equation were analyzed for removal at each step, using a p > 0.05 significance level. The stepwise process was halted when the residual chi-square was no longer significant.

The final model was evaluated for, (i) goodness of fit (deviance statistic), (ii) the association of predictor variables with the outcome variable (Nagelkerke's R^2^), (iii) success in classification (% correctly classified cases, sensitivity and specificity), and (iv) the discriminatory power of the logistic equation (*c *statistic), see Table 1.

Individual parameters in each model are presented as odds ratios for categorical data and p-deviance for continuous variables. To aid interpretation, on occasions when the odds ratio was less than one, its reciprocal was taken and the finding interpreted in terms of the odds regarding the reference group.

## Results

### Preliminary group differences

#### Demographic factors

The sample consisted of 175 males and 39 females, with similar proportions of males and females in each treatment group (CABG: 66 males, 7 females; PTCA: 57 males, 14 females; medication: 52 males, 18 females; χ^2 ^= 6.394, df = 2, p = 0.041; φ_c _= 0.173). The mean age of the sample was 64.1 years, with no age differences between treatment groups (f_(2,213) _= 2.588, p = 0.078; ω^2 ^= 0.015). The employment status of patients did not significantly differ between groups (χ^2 ^= 19.967, df = 12, p = 0.068; φ_c _= 0.216). The PTCA participants were found to have a higher educational level than the other two groups (Brown-Forsythe, f_(2,202.030) _= 10.605, p < 0.001; ω^2 ^= 0.082; PTCA mean = 2.51, medication mean = 1.97, CABG mean score = 1.64).

#### Cardiac history, risk factors and co-morbidities

The three treatment groups were relatively similar with regards co-morbidity, risk factors and cardiac history. Only three variables differed significantly between the groups. The medication group had the highest rate of cerebral events (n = 12), followed by the PTCA (n = 7) then the CABG (n = 1; χ^2 ^= 10.526, df = 2, p = 0.005; φ_c _= 0.222); however, the overall frequency of events was modest. The PTCA group had more previous interventions than the other two groups (χ^2 ^= 27.779, df = 2, p < 0.001; φ_c _= 0.360), the majority of which were previous PTCA (13/25 PTCA alone; 8/25 CABG alone; 4/25 PTCA and CABG). Rates of family history of IHD also varied significantly between groups (χ^2 ^= 11.393, df = 2, p = 0.003; φ_c _= 0.231). PTCA (n = 34) patients had lower rates than the medication (n = 51) and CABG (n = 51) groups. Rates of other conditions were similar between treatments groups, and the common risk factors for CAD did not significantly differ between groups.

#### Angiogram reports

Objective disease scores revealed significant treatment group effects (p < 0.01) for all indices except right coronary artery disease (f_(2,111) _= 4.319, p = 0.015; ω^2 ^= 0.030). LAD artery disease (Brown-Forsythe, f_(2,184.061) _= 31.712, p < 0.001; ω^2 ^= 0.223), significantly differed between medication and the two revascularisation groups. Circumflex artery scores (Brown-Forsythe, f_(2,201.629) _= 19.785, p < 0.001; ω^2 ^= 0.149), total vessels diseased scores (Brown-Forsythe, f_(2,183.301) _= 6.525, p = 0.002; ω^2 ^= 0.049), and ventricular function scores (Brown-Forsythe, f_(2,183.046) _= 8.533, p < 0.001; ω^2 ^= 0.066), all produced a pattern of group differences where the medication and PTCA groups significantly differing from the CABG group but not from each other. Group mean scores are shown in Table 2.

**Table 2:** Demographics, angiogram scores, symptom reports and functional ability by treatment group

		Total	Medication	PTCA	CABG	p
		214	70	71	73	
Gender	Male	175	52	57	66	
	Female	39	18	14	7	0.041
						
Age	Mean	64.09	66.00	62.03	64.26	0.078
Education level	Mean	2.04	1. 97^a^	2.51^b^	1.64^a^	<0.001

Angiogram scores	LAD artery disease	2.18	1.56^a^	2.31^b^	2.64^b^	<0.001
	Circumflex artery disease	1.67	1.30^a^	1.39^a^	2.30^b^	<0.001
	RCA disease	1.98	1.80	1.85	2.27	0.015
	Number of disease vessels	2.51	2.39^a^	2.37^a^	2.77^b^	0.002
	Ventricular Function score	0.63	0.56^a^	0.39^a^	0.93^b^	<0.001

Symptom frequency	Angina	2.03	1.80^a^	2.18^b^	2.11^b^	0.005
	Dyspnoea	2.22	2.19	2.37	2.11	0.212
	Non-cardiac	1.43	1.38	1.46	1.46	0.196

Functional limitations	Angina associated	1.80	1.29^a^	1.93^b^	2.15^b^	<0.001
	Dyspnoea associated	1.56	1.21	1.67	1.79	0.013

Activities of daily living	Social Behaviour	4.43	3.96^a^	3.97^a^	5.32^b^	0.005
	Emotional Stability	1.30	1.43	1.48	1.00	0.112
	Mobility Range	1.91	1.00^a^	1.15^a^	3.52^b^	<0.001
	Somatic Autonomy	0.84	0.94	0.58	0.99	0.200
	Mobility Control	4.00	3.81	3.63	4.53	0.143
	Psychological Autonomy & Communication	1.71	1.89	1.75	1.51	0.613

Gender analyses conducted with Chi-sq test, remainder with ANOVAs.

Significant treatment group differences revealed within the ANOVAs are indicated with different superscript letters.

#### Cardiac symptoms and associated functional limitations

Significant treatment group differences were found on the angina frequency scores, (f_(2,211) _= 5.444, p = 0.005; ω^2 ^= 0.040). Post hoc tests revealed the medication group to have significantly less frequent angina than the PTCA and CABG groups. The frequency of shortness of breath did not significantly vary between treatment groups (f_(2,211) _= 1.562, p = 0.212; ω^2 ^= 0.005); nor did the level of non-cardiac symptoms (f_(2,211) _= 1.640, p = 0.196; ω^2 ^= 0.006).

One-way ANOVAs of the functional limitations associated with cardiac symptoms, revealed that the ratings for angina were significantly different between treatment groups (Brown-Forsythe, f_(2,204.159) _= 9.618, p < 0.001; ω^2 ^= 0.075), but not for dyspnoea (Brown-Forsythe, f_(2,201.311) _= 4.474, p = 0.013; ω^2 ^= 0.031). The post hoc tests for angina ratings showed the medication group was significantly less functionally impaired by angina than both the PTCA and CABG patients.

#### Activities of daily living

On limitations in activities of daily living, the groups differed significantly on two indices, social behaviour (f_(2,211) _= 5.398, p = 0.005; ω^2 ^= 0.039) and mobility range (Brown-Forsythe, f_(2,185.686) _= 33.072, p < 0.001; ω^2 ^= 0.231). In both cases, the medication and PTCA groups were significantly different from the CABG group but not from each other. The other activity indices revealed similar group scores: Emotional Stability, (Brown-Forsythe, f_(2,197.489) _= 2.211, p = 0.112; ω^2 ^= 0.011); Somatic Autonomy, (f_(2,211) _= 1.612, p = 0.200; ω^2 ^= 0.006); Mobility Control, (f_(2,211) _= 1.963, p = 0.143; ω^2 ^= 0.009); and Psychological Autonomy and Communication, (f_(2,211) _= 0.490, p = 0.613; ω^2 ^= 0.000).

### Predictors of treatment group

The univariate analyses (Table 3) were followed by the same comparisons in a multivariate analysis (Table 4). The results of both sets of analyses are collated and summarised in Table 5.

**Table 3:** Variables that achieved significance in the univariate comparisons between treatment groups

VARIABLES	Likelihood ratio			Exp (β)	Confidence interval		RATES/MEAN SCORES	
	
	Model change	df.	Sig.	Odds Ratio	Lower bound	Upper bound	Reference	Response
(a) PTCA from Medication							Medication	PTCA
Increasing age	4.644	1	0.031	0.967	0.928	1.008	66.00	62.03
Higher educational attainment	6.887	1	0.009	1.447	0.999	2.096	1.97	2.51
Previous intervention	20.517	1	< 0.001	8.961	2.055	39.067	4	25
Hypertension	4.505	1	0.034	0.482	0.197	1.178	36	24
Family history: IHD	9.306	1	0.002	0.342	0.136	0.863	51	34
LAD artery disease	21.166	1	< 0.001	2.328	1.392	3.893	1.56	2.31
Angina frequency	11.204	1	0.001	2.351	1.182	4.677	1.80	2.18
Angina functional limitations	11.079	1	0.001	1.651	1.102	2.474	1.29	1.93
Dyspnoea functional limitations	5.851	1	0.016	1.444	0.968	2.153	1.21	1.67

(b) CABG from PTCA							PTCA	CABG
Higher educational attainment	19.186	1	< 0.001	0.525	0.350	0.786	2.51	1.64
Previous intervention	16.410	1	< 0.001	0.165	0.046	0.587	25	6
Family history: IHD	7.251	1	0.007	2.523	1.028	6.192	34	51
Family history: Neurological	5.400	1	0.020	0.194	0.025	1.527	9	2
Cerebral events	5.505	1	0.019	0.127	0.008	2.065	7	1
LAD artery disease	6.996	1	0.008	1.834	0.980	3.435	2.31	2.64
Circumflex artery disease	22.489	1	< 0.001	2.058	1.352	3.132	1.39	2.30
RCA disease	5.600	1	0.018	1.444	0.960	2.173	1.85	2.27
Number of vessels diseased	12.709	1	< 0.001	2.546	1.221	5.310	2.37	2.77
Ventricular disease	12.979	1	< 0.001	2.037	1.172	3.540	0.39	0.93
SIP: Social behaviour	7.937	1	0.005	1.182	1.010	1.382	3.97	5.32
SIP: Mobility range	35.312	1	< 0.001	1.608	1.254	2.062	1.15	3.52

(c) CABG from Medication							Medication	CABG
Gender	6.612	1	0.010	0.306	0.088	1.062	18	7
Hypercholesterolemia	6.892	1	0.009	0.410	0.169	0.994	45	31
Hypertension	6.773	1	0.009	0.407	0.166	1.003	36	22
Cerebral events	12.419	1	< 0.001	0.067	0.004	1.018	12	1
LAD artery disease	54.350	1	< 0.001	5.396	2.553	11.407	1.56	2.64
Circumflex artery disease	32.294	1	< 0.001	2.647	1.617	4.333	1.30	2.30
RCA disease	7.800	1	0.005	1.599	1.024	2.495	1.80	2.27
Number of vessels diseased	9.668	1	0.002	2.116	1.080	4.144	2.39	2.77
Ventricular disease	7.173	1	0.007	1.737	0.998	3.023	0.56	0.93
Angina frequency	5.824	1	0.016	1.710	0.949	3.080	1.80	2.11
Angina functional limitations	15.130	1	< 0.001	1.660	1.168	2.358	1.29	2.15
Dyspnoea functional limitations	7.619	1	0.006	1.450	1.015	2.072	1.21	1.79
SIP: Social behaviour	7.714	1	0.005	1.175	1.008	1.371	3.96	5.32
SIP: Mobility range	40.346	1	< 0.001	1.683	1.299	2.181	1.00	3.52

**Table 4:** Multivariate analysis comparing pairs of treatment groups

	Analysis of maximum likelihood estimates							odds ratio/p-deviance		
Parameter	Entry	df	estimate	std. error	Wald χ^2^	Pr > chi square	std. estimate	point estimate	99% Wald CI	99% Wald CI
(a) Medication vs. PTCA										
Constant	0	1	1.131	1.454	0.606	0.437	.			
LAD artery disease	1	1	0.970	0.240	16.374	< 0.001	0.536	2.639	1.423	4.894
Previous intervention	2	1	1.093	0.322	11.536	0.001	.	8.896	1.696	46.675
Increasing age	3	1	-0.065	0.021	9.364	0.002	-0.397	0.937	0.887	0.990
Hypertension	4	1	-0.664	0.229	8.373	0.004	.	0.265	0.081	0.864
Angina frequency	5	1	0.883	0.341	6.694	0.010	0.337	2.418	1.004	5.822

(b) PTCA vs. CABG										
Intercept	0	1	-1.889	0.710	7.091	0.008	.			
SIP: Mobility range	1	1	0.475	0.112	17.868	< 0.001	0.667	1.607	1.204	2.147
Circumflex artery disease	2	1	0.756	0.215	12.345	< 0.001	0.490	2.128	1.223	3.704
Previous intervention	3	1	-1.246	0.337	13.674	< 0.001	.	0.083	0.015	0.469
Higher educational attainment	4	1	-0.661	0.204	10.545	0.001	-0.441	0.516	0.305	0.872

(c) Medication vs. CABG										
Intercept	0	1	-8.831	1.548	32.561	< 0.001	.			
LAD artery disease	1	1	1.616	0.373	18.804	< 0.001	0.853	5.034	1.927	13.150
Previous cerebral events	2	1	-2.463	0.707	12.127	0.001	.	0.007	0.000	0.277
SIP: Mobility range	3	1	0.620	0.162	14.761	< 0.001	0.870	1.859	1.227	2.818
Circumflex artery disease	4	1	1.095	0.293	13.991	< 0.001	0.661	2.991	1.406	6.359

**Table 5:** Summary findings of univariate and multivariate significant effects

Univariate	PTCA from Medication	CABG from PTCA	CABG from Medication
Increasing age	▼		
Gender			▼
Higher educational attainment	▲	▼	
mployment			

Previous coronary intervention	▲	▼	
Hypercholesterolemia			▼
Hypertension	▼		▼
Previous MI			
Diabetes			
Smoking history			

Family history: IHD	▼	▲	
Family history: Neurological		▼	
Family history: MI			
Family history: Stroke			

Previous cerebral events		▼	▼
Arthritis/Rheumatism			
Respiratory			
Renal complications			
GI tract problems			
Peripheral vascular disease			
Thyroid problems			
Varicose veins			

LAD artery disease	▲	▲	▲
Circumflex artery disease		▲	▲
RCA disease		▲	▲
Number of vessels diseased		▲	▲
Ventricular disease		▲	▲

Angina frequency	▲		▲
Dyspnoea frequency			
Non-cardiac symptoms			
Angina functional limitations	▲		▲
Dyspnoea functional limitations	▲		▲
SIP: Social behaviour		▲	▲
SIP: Mobility range		▲	▲
SIP: Emotional stability			
SIP: Somatic autonomy			
SIP: Mobility control			

Multivariate	PTCA from Medication – 5	CABG from PTCA – 4	CABG from Medication – 4

Increasing age	▼**3**		
Higher educational attainment		▼**4**	

Previous coronary intervention	▲**2**	▼**3**	
Hypertension	▼**4**		

Previous cerebral events			▼**2**

LAD artery disease	▲**1**		▲**1**
Circumflex artery disease		▲**2**	▲**4**

Angina frequency	▲**5**		
SIP: Mobility range		▲**1**	▲**3**

▲ = increase in odds; ▼ = decrease in odds; numerals indicate order of entry during the stepwise procedure

#### Analyses I: PTCA compared to medication

In the univariate analysis, nine of the predictor variables produced significant findings (see Table 3a). Increasing age, hypertension and a family history of ischemic heart disease (IHD) reduced the odds of a patient receiving PTCA. Educational level, previous interventions, angina and dyspnoea related functional limitations, angina frequency and LAD disease, increased the odds of PTCA.

Within the multivariate analysis (Table 4a), LAD artery disease, previous intervention, age, hypertension and angina frequency were included in the final model. The overall model produces a satisfactory goodness of fit, with non-significant residual variance (deviance = 135.184, df = 135, p = 0.479). Nagelkerke's R^2 ^= 0.464, indicated the predictor variables were associated reasonably well with the outcome variable. Correct classification of cases moved from 50.4% to 70.2% in the final model, with 71.8% of PTCA patients (i.e. sensitivity) and 68.6% of medication patients correctly classified (i.e. specificity). The discriminatory power of the model is fairly strong with the c-statistic at 0.849.

Examination of parameter estimates for categorical predictors in this model indicated that holding other variables constant, (i) having had a previous cardiac intervention increased the odds of PTCA by ≈ 9 times, and (ii) hypertension increased the odds of being in the medication group by ≈ 4 times. On the continuous predictors, the odds of being a medication patient increased by ≈ 7% for each years increase in age. In this instance, an increment of 10 years in age doubled the odds of being in the medication as opposed to PTCA group. Unsurprisingly, greater angina frequency and LAD artery disease both increased the odds of having PTCA. Thus going from '*occasional*' angina to '*frequent*' angina increased the odds of PTCA by 2.4 times and going from '*mild*' to '*moderate*' levels of LAD artery disease increased the odds of PTCA by 2.6 times.

#### Analyses II: CABG compared to PTCA

Univariate analyses identified twelve variables as significant predictors of CABG treatment as opposed to PTCA (see Table 3b). A higher level of education, having received previous cardiac interventions, family history of neurological conditions and previous cerebral events were all associated with lowered odds of receiving CABG. Higher scores on the five indices from the angiogram, a family history of IHD and reduced levels of social behaviour and mobility range (SIP-68) resulted in greater odds of CABG. Four of these univariate predictors were retained in the final stepwise multivariate model: education level, previous cardiac intervention, circumflex artery disease and mobility range (see Table 4b). This model was satisfactory with regards to overall model fit, with non-significant residual variance (deviance = 118.363, df = 138 p = 0.885), and Nagelkerke's R^2 ^at 57.5%. Classification of cases moved from 50.7% to 78.5% correct in the final model; 78.1% of CABG patients and 78.9% of PTCA patients were correctly classified. The discriminatory power of the logistic equation was relatively good (c = 0.892).

The individual parameter estimates indicated that the odds for PTCA increased by ≈12 times if a previous intervention had been undertaken. The continuous predictors revealed that a unit increase in educational level (e.g. going from A-level to graduate standard) almost halved the odds of being in the CABG group. A single additional score on the functional limitation on the SIP mobility range scale increased the odds of CABG by 60% and moving from '*mild*' to '*moderate*' levels of circumflex artery disease increased the odds of CABG by ≈ 2 times.

#### Analyses III: CABG compared to medication

Fourteen predictors, listed in Table 3c, were significant in the univariate analyses. Increasing severity within all the angiogram and function related scores significantly increased the likelihood of receiving a CABG. The presence of co-morbidities (hypercholesterolemia, hypertension and previous cerebral events) and being female individually reduced the likelihood of CABG.

The multivariate stepwise analysis of these predictors indicated relatively little residual variance (deviance = 85.691, df = 138, p = 0.999), a high association between the DV and the predictors (Nagelkerke's R^2 ^= 0.726), and a large proportion of correctly classified cases (84.6%, up from 51.0% in the constant only model; sensitivity = 84.9%; specificity = 84.3%). The discriminatory power of the logistic regression equation was the highest of the three sets (c = 0.944).

Having previous cerebral events increased the odds of receiving medication treatment by 142.9 times, (see Table 4c). On the continuous measures, the odds of CABG were ≈ 5 times higher with a one unit increase in LAD artery disease and ≈ 3 times higher with unit increases in circumflex artery disease severity. Greater mobility range limitations increased the odds of CABG by ≈ 2 times per unit increase.

## Discussion

This study examined factors that influenced the allocation of patients to different treatments for CAD. Three sets of analyses were conducted comparing actual decisions to undergo: PTCA rather than receive medication, CABG rather than PTCA, and CABG rather than medication.

Univariate analyses revealed a wide range of differentiating factors including, demographic variables, co-morbidities, family histories, symptom reports, functional limitations and angiogram indices (i.e. objective disease reports). While many of these factors are in the treatment guidelines (e.g. co-morbidities, angiogram indices); others are not (e.g. educational level, patient rated functioning or symptom reports), but appear to be, on empirical analysis, implicitly involved in the decision processes, particularly in analyses involving comparisons with PTCA. This indicates that clinicians are undoubtedly aware that multiple factors outside of those explicitly discussed in guidelines (which are usually technical and clinical in nature), should be addressed and guide decision making about treatment modality.

Gender has long been a controversial issue in CAD and much evidence has accumulated regarding poorer outcomes for women [16]. Research investigating, gender differences in patients undergoing evaluation for CAD has found contradictory results but generally suggested that resource utilisation may be lower in women [17-19]. In a large study, Miller et al. [20] did find less coronary angiographies performed in women being assessed for CAD and lower referral for revascularisation in univariate analysis. Although, in this study, females were almost 70% less likely than males to have CABG when compared to medication, this effect was not robust enough to be retained in the multivariate analysis, suggesting that gender is not a primary concern during this decision process, between the two most disparate treatments. Further, in the other analyses there was no evidence to suggest a bias for males to receive particular interventions. However, these findings must be interpreted in light of the small number of female participants, which may limit the power to find gender effects.

Previous research has shown that mortality increases with age in both CABG and PTCA [21,22]. In the present study age was not a significant factor in determining CABG from the other two treatments. The CABG results were unexpected but the increased risks may have been counter-balanced by judgements regarding graft patency and life expectancy. In contrast, an increase of ten years in age nearly doubled the likelihood of medication over PTCA in the multivariate analysis. While this may reflect a natural tendency to perform less invasive procedures in the elderly, it is also important that the PTCA group had significantly higher levels of education in comparison to both medication and CABG groups. One may speculate that the younger more educated individuals may be both more aware of and willing to try newer treatments and possibly more actively involved in their treatment decisions. It is however important that the PTCA group were also distinguished by their greater experience of CAD treatments and possible vicarious knowledge/experience from family members who had a history of IHD. PTCA patients were more likely to have received a previous intervention (mainly PTCA's) than both the medication and CABG patients in both the univariate and multivariate analyses. Given the relatively high rates of restenosis following PTCA, this finding is not unexpected. The previous experiences may have provided patients with clearer expectations upon which to base and participate in the decisions regarding their treatment.

Variables concerning patient history and co-morbidities appeared to appropriately reduce the likelihood of the more interventional treatments. Hypertension had a tendency to increase the odds of remaining on/starting medication. In the PTCA versus medication analysis, this effect was retained in the multivariate analysis. Hypercholesterolemia reduced the likelihood of CABG as opposed to medication, and the effects of a family history of neurological problems reduced the likelihood of CABG from PTCA. Less obviously a family history of IHD, reduced the odds of PTCA when compared to both medication and CABG. However, none of these findings were retained in the multivariate analyses.

The measures from the angiogram reports demonstrated a strikingly consistent effect in predicting the likelihood of CABG, as would be expected and is supportive of the guidelines. Within the univariate analysis, all five of the measures (levels of LAD, circumflex and right coronary artery disease, the number of disease arteries and the level of ventricular disease) increased the likelihood of CABG. The per unit increase in these measures led to a multiplication in odds of CABG ranging from 1.6 to 5.4 times in the medication versus CABG analyses, and between 1.5 and 2.5 times for the PTCA versus CABG analysis. For the most part, the odds ratios determining CABG from medication were larger than those determining CABG from PTCA, as would be expected. The other significant result from the univariate analyses of the angiogram data showed that increasing LAD artery disease predicts a greater likelihood of PTCA from medication; a finding consistent with treatment guidelines.

The multivariate analyses helped determine which of the angiogram measures was the most relevant to the treatment decision process. The pattern of results indicated that with increased LAD disease, medication was not considered a sufficient treatment; the likelihood of PTCA or CABG was increased. Moving from mild to moderate LAD disease increased the odds of PTCA from medication by ≈ 2.6 times, and the odds of CABG from medication by approximately twice this (5 times). Increasing LAD disease did not significantly alter the odds of CABG in comparison to PTCA.

Increasing levels of disease in the circumflex artery increased the likelihood that CABG would be the chosen option. A move from mild to moderate levels of circumflex artery disease more than doubled the odds of CABG from PTCA and almost trebled the odds of CABG as opposed to medication. Interestingly, the number of diseased arteries, which is often an indication for CABG rather than PTCA [6], was not retained in the multivariate model. These results are reassuring in that they confirm the important role of the angiogram reports when deciding whether to proceed with interventional strategies, in particular CABG.

Patient reports of the frequency of their symptoms, and the manner in which their poor health is affecting their lives, are the customary way in which patients present the condition to their health care professionals. High levels of angina symptom reports and greater functional limitations increased the likelihood of receiving either of the two more invasive procedures. They did not, however, significantly affect the decision between PTCA and CABG. The symptom reports that appeared to drive the decisions were specific and non-cardiac symptoms did not affect the treatment decision. In the multivariate analysis, the frequency of angina was retained when comparing PTCA to medication, such that moving from occasional to frequent angina increased the odds of PTCA by ≈ 2.4 times. The finding that only one symptom measure entered each multivariate analysis is likely to be due to the degree of correlation between the various symptom report measures (*range from 0.731 to 0.225 all p < 0.001*).

Reports of restrictions on activities of daily living were also found to influence treatment decisions. Specifically, greater limitations in social behaviours and mobility increased the likelihood of receiving CABG, from both PTCA and medication. When entered into the multivariate analysis, the illnesses effect on mobility was the only measure of activities of daily living that was retained; increasing the odds of CABG from medication and PTCA by 80% and 60% respectively, for a unit increase in mobility range problems. This suggests that mobility restrictions are an important trigger to provoke more invasive treatments by cardiologists.

Overall each category of information was represented within the multivariate analyses, although all the categories were not represented in each of the analyses. This suggests that all types of information are being considered during the decision processes, although some may only be evident when examined within specific treatment comparisons. Two distinct patterns of outcomes can be discerned from the cumulative results of the three sets of analyses. First, the increased likelihood of CABG was primarily based on reports of greater objective disease (angiograms) and functional limitations. Second, the data suggests that decisions concerning PTCA can be interpreted to be driven by patient characteristics which may reflect the influence of patient preferences although these were not directly measured in this study. The findings reveal that treatment decisions are subject to a range of different influences and not simply guided by protocols.

It must be noted that treatment modalities/technologies and the guiding protocols are changing constantly. The management of coronary disease has changed over the period since the participants for this study were recruited (e.g. as many as three times more percutaneous coronary interventions are now done in the UK). However, although the manner in which factors are addressed in allocating patients to treatments modalities during the decision making process has altered in guidelines, the specific factors that are considered have not changed significantly in the recent updates [4,6].

Potential limitations to this study include the relatively small sample of participants, which were predominately male and drawn from a limited geographical region, taken from a small pool of cardiologists; resulting in a highly selective patient population (with low incidences of some co-morbidities). Each of these factors may limit the generalisability of results (e.g. the small number of female participants may have precluded finding significant gender effects). A larger multi-centre study, with stratified sampling may help to resolve some of these issues, and it is acknowledged that both geographical and individual consultant factors may confound treatment decisions. For instance there may be a proclivity to perform interventional cardiology in certain regions and/or that individual cardiologists may be referred particular patients, and have preferences for particular treatments [23]. More detailed analysis of such factors with a larger study sample may help to unravel how such factors confound treatment decision processes.

Additionally, a limitation of observational studies, such as the current one, is that they cannot exclude as explanations other unmeasured factors [11]. It may be necessary to conduct further studies to see how recent changes in treatment and technology effects decision making. This study provides both a basis for this and evidence that treatment decisions are subject to a range of different influences including those in protocols.

## Conclusion

Although there are a number of clinical guidelines for deciding between the treatments available for CAD, this study shows that variables beyond those in these protocols, such as patient reports of disability and patient characteristics, may also be important variables when therapy decisions are undertaken. The role of these factors and their relationship and interactions with factors emphasised in the guidelines require further investigation to delineate the processes involved in treatment decisions for CAD.

## Abbreviations

ANOVA – analysis of variance

CABG – coronary artery bypass grafting surgery

CAD – coronary artery disease

IHD – ischemic heart disease

LAD – left anterior descending

MCAR – missing completely at random

PTCA – percutaneous transluminal coronary angioplasty

RCA – right coronary artery

SIP – sickness impact profile

## Competing interests

The author(s) declare that they have no competing interests.

## Authors' contributions

SPH & SPN contributed to the development and design of the study

SPH, SPN, JAH & SS contributed to the analysis of the data

All authors contributed to the writing and reviewing of the manuscript

## Pre-publication history

The pre-publication history for this paper can be accessed here:


